# The impact of an intensive care unit admission on the health status of relatives of intensive care survivors: A prospective cohort study in primary care

**DOI:** 10.1080/13814788.2022.2057947

**Published:** 2022-04-07

**Authors:** Rick Naaktgeboren, Marieke Zegers, Marco Peters, Reinier Akkermans, Hans Peters, Mark van den Boogaard, Floris A. van de Laar

**Affiliations:** a Department of Primary and Community Care, Radboud University Medical Center, Nijmegen, The Netherlands; b Department of Intensive Care, Radboud Institute for Health Science, Radboud University Medical Center, Nijmegen, The Netherlands; c Department of Intensive Care, Canisius Wilhelmina Ziekenhuis, Nijmegen, The Netherlands

**Keywords:** General practice, intensive care unit, family, post-intensive care syndrome, posttraumatic stress symptoms

## Abstract

**Background:**

Relatives of intensive care unit (ICU) survivors may suffer from various symptoms after ICU admittance of their relative, known as post-intensive care syndrome-family (PICS-F). Studies regarding PICS-F have been performed but its impact in primary care is unknown.

**Objectives:**

To explore health problems of relatives of ICU survivors in primary care.

**Methods:**

This is an exploratory prospective cohort study in which we combined data from two hospitals and a primary care research network in the Netherlands. ICU survivors who had been admitted between January 2005 and July 2017 were identified and matched by sex and age with up to four chronically ill (e.g. COPD, cardiovascular disease) patients. In both groups, relatives living in the same household were identified and included in this study. Primary outcome was the number of new episodes of care (International Classification of Primary Care-2) for up to five years. Hazard ratios (HRs) for the total number of new episodes were calculated.

**Results:**

Relatives of ICU survivors (*n* = 267, mean age 38.1 years, 41.0% male) had significantly more new care episodes compared to the reference group (*n* = 705, mean age 36.3 years, 41.1% male) 1–2 years (median 0.11 vs. 0.08, HR 1.26; 95% confidence interval (CI) 1.03–1.54) and 2–5 years (median 0.18 vs. 0.13, HR 1.28; 95%CI 1.06–1.56) after ICU discharge. No differences were found in the period before ICU admission.

**Conclusion:**

Relatives of ICU survivors present more morbidity in primary care than relatives of chronically ill patients up to five years after ICU discharge.

KEY MESSAGES

- Relatives of ICU survivors had significantly more new episodes of care in primary care the second to the fifth year after ICU admission than relatives of chronically ill patients.
- GPs should examine the role ICU admission might play in current health problems in relatives of ICU survivors.

## Introduction

In the Netherlands annually, over 80,000 critically ill patients are admitted to the intensive care unit (ICU), of whom more than 90% survive [1]. The total number but also the survival rate and mean age of these ICU patients is ever increasing [2,3]. Despite these successes, ICU survivors may develop new or worsening impairments in physical, cognitive or mental functioning after discharge from the ICU. This cluster of illnesses after ICU treatment is referred to as the post-intensive care syndrome (PICS) [4,5], which may subsequently result in increased use of primary care [6,7].

In addition, relatives of ICU survivors may also experience psychological and emotional distress. They may suffer from sleep deprivation [8], anxiety, stress and worries about the health and treatment of their loved one or finances [9]. These symptoms can last for years after ICU discharge and are referred to as the post-intensive care syndrome-family (PICS-F) [10–16]. Health problems observed in PICS-F are depression, anxiety, posttraumatic stress disorders, a decrease in health-related quality of life and fatigue [17,18].

These PICS-F related symptoms may lead to increased consultations in primary care. Consequently, general practitioners (GPs) would need to be extra vigilant regarding this group of patients, especially when we are seeing a significant increase in numbers of ICU patients due to COVID-19. However, to date, little is known about the occurrence of PICS-F in primary care, the frequency of visitations and the nature of health problems presented to the GP. Furthermore, GPs and GP staff lack the knowledge and resources to improve care for ICU survivors and their relatives [19]. Moreover, it is unknown whether this is a typical ICU related problem or comparable to the problems relatives of patients with a chronic illness face [9].

Therefore, the primary aim of this study was to explore the number of health episodes among relatives of ICU survivors presented in primary care and compare the number of health episodes to those among relatives of patients with a chronic disease; furthermore, to determine differences in the number of health episodes clustered by diagnosis group and pathophysiology and in the total number of GP consultations between these groups.

## Methods

### Design and data source

This is a prospective cohort study using both primary care and hospital care data. Primary care data were derived from Family Medicine Network (FaMe-net): a primary care research network, which is subject to systematic quality tests [20–22]. The network comprises nine general practices in the Netherlands that prospectively and structurally register diagnoses according to the International Classification of Primary Care-2 (ICPC-2) coding system [23,24]. For this study, we used data from three practices adjacent to the two participating ICUs. Hospital care data were derived from Epic (Radboud University Medical Center) and HiX (Canisius Wilhelmina Ziekenhuis). Information extracted from the Fame-Net database is stored secure and de-identified according to Dutch privacy legislation. Patients listed in the practices may opt-out to extract their data for research [21].

### Index group

We identified all patients that were admitted between January 2005 and July 2017 to two Dutch ICUs (Radboud University Medical Centre and Canisius Wilhelmina Ziekenhuis), and were listed in one of the three participating FaMe-net general practices. Demographic data, comorbidities, length of ICU stay and APACHE-II scores of the ICU survivors were collected. Next, all relatives of all ages who resided in the same household as the ICU survivors during the ICU admission were identified. Relatives of ICU survivors were referred to as index relatives. Follow-up period was defined as the time between ICU discharge of the ICU survivor related to the index relative and the five-year point after ICU discharge, after which we stopped collecting data.

### Reference group

ICU survivors were meticulously matched for gender, 10-year interval age group and pre-existent comorbidity with chronically ill patients but had no history of ICU stay. These chronically ill patients suffered from various diseases such as cancer, COPD and cardiovascular disease (Supplementary Table 1). Comorbidity was considered pre-existing when it was registered before the date of the matched ICU hospitalisation of the ICU survivor [25]. For the reference group, all relatives of all ages who resided in the same household as the matched chronically ill patients were identified. Relatives of chronically ill patients were referred to as reference relatives.

### Matching procedure

**Figure 1.**
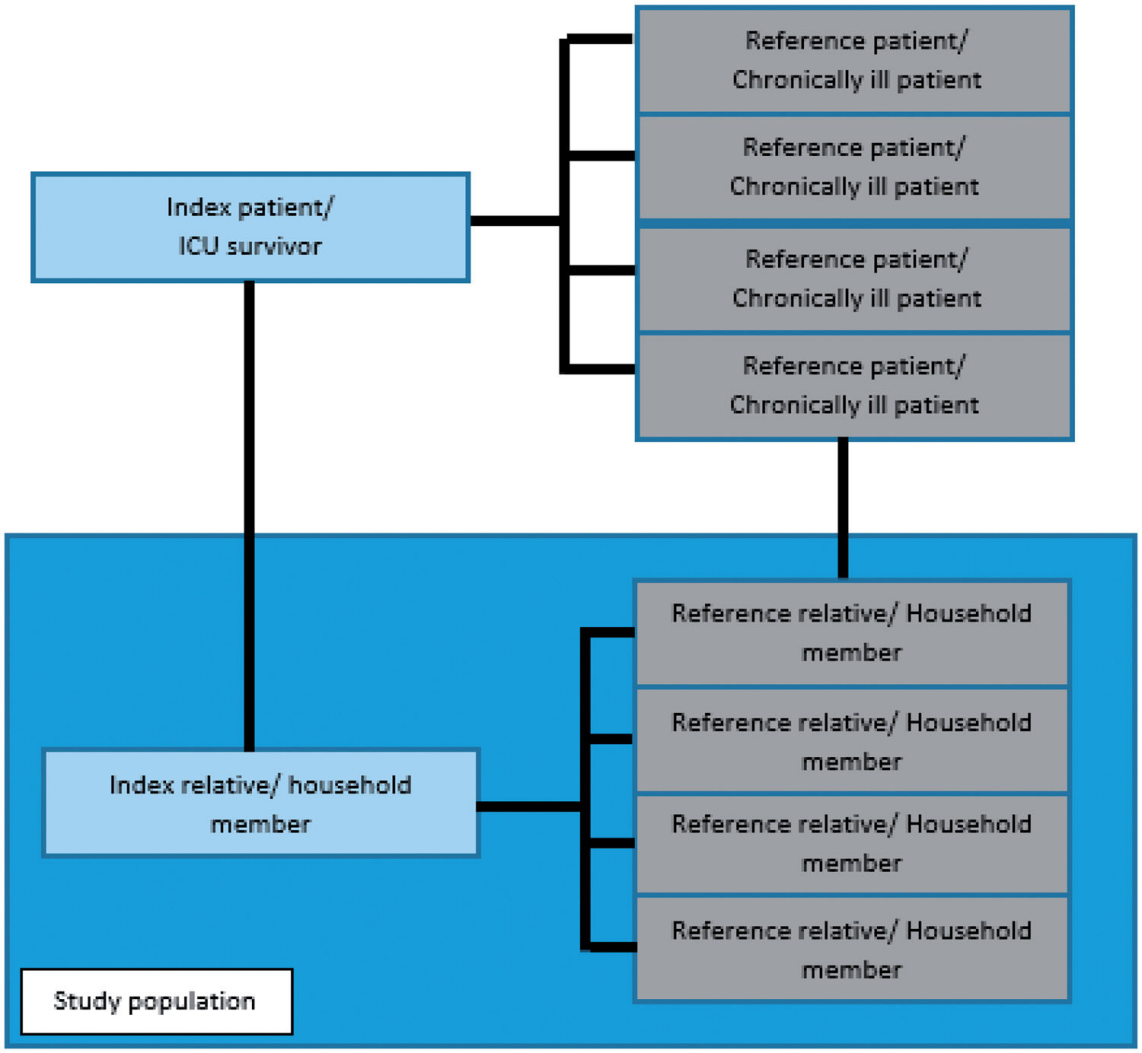
Flowchart of matching procedure: ICU survivors were meticulously matched for gender, 10-year interval age group and pre-existent comorbidity with patients who were chronically ill but had no history of ICU stay. For the reference group, all relatives of all ages who resided in the same household as the matched chronically ill patients were identified. Relatives of chronically ill patients were referred to as reference relatives. Index relatives and reference relatives were compared based on matching ICU patient to a corresponding chronic ill patient. To increase the power of the study, one index relative was compared with up to four reference relatives.

Index relatives and reference relatives were compared based on matching ICU patients to a corresponding chronically ill patient. To increase the power of the study, one index relative was compared with up to four reference relatives. The matching procedure is graphically displayed in Figure 1. Households of patients who resided alone were excluded. Households with multiple chronically ill patients or households with a chronically ill patient and ICU survivor were also excluded due to the risk of effect modifying and confounding.

### Pre- and post-ICU timeframes

A total of six timeframes were defined for this study: 0–1 year before ICU admission and 0–3, 3–6 and 6–12 months, and 1–2 and 2–5 years after ICU discharge. Pre-ICU timeframe was chosen to create a baseline for the two cohorts by comparing the total number of new episodes of care (episodes) and consultations between index and reference relatives one year prior the follow-up period. Post-ICU timeframes were chosen because symptoms of PICS-F may develop quickly after ICU discharge and can persist up to five years [16]. Index and reference relatives were included in each timeframe. To better compare the different timeframes, we additionally merged the first-year follow-up as one timeframe of 0–12 months in the analysis of the ICPC-2 chapters.

### Outcome measures

The primary outcome was the number of new episodes. An episode is a single or a series of encounters with the healthcare provider about one defined health problem. In the Dutch healthcare system, episodes initiated by specialist care, by other primary care health providers or by out-of-hours services are reported back to the GP and were as such, also counted as new episodes. During an episode, the diagnosis may evolve depending on the development of the problem and the considerations of the healthcare provider. For each episode, the ‘final’ diagnosis was retrieved as coded by ICPC-2, enabling coding on ‘symptom level’ and on ‘diagnosis level.’ Secondary outcome measures were the number of new episodes clustered by ICPC-2 chapter (organ system e.g. respiratory, skin) and ICPC-2 pathophysiologies (e.g. infections, injuries), and the total number of GP consultations clustered by type of consultation (e.g. phone calls and visits).

### Data analysis

Categorical variables were presented as a number stating frequency with percentage, and continuous variables as a mean ± standard deviation (SD) when distributed normally or as median with first and third quartile, expressed as interquartile range (IQR) when skewed distributed. Hazard ratios (HRs) with 95% confidence intervals (95%CI) were calculated for total number of new episodes, new episodes clustered by ICPC-2 chapter and pathophysiology and consultations associated with having a relative in the ICU using a negative binomial regression model. The negative binomial regression model was chosen because overly skewed count data were analysed. Our outcome measures were corrected for age, gender, general practice of the relative and early or late discharge of ICU survivor and statistically significant difference in HR in the pre-ICU timeframe. To reduce multiple testing and due to deficient numbers of new episodes of care in certain ICPC-2 chapters, we merged the first-year follow-up as one timeframe of 0–12 months for the analyses of the episodes clustered by ICPC-2 chapter. All outcomes were compared with the matching measures in the reference relatives in each timeframe. Independent samples *t*-test was used to compare the mean values and chi-squared test was used to compare frequencies between index and reference group. Statistical significance was set at *p* < .05 for all analyses. Data analysis was performed with IBM SPSS Statistics 25.0 (Chicago, IL).

## Results

### Study population

The index and reference groups in our research were the relatives of respectively 153 ICU survivors (median ICU stay of one day, (IQR 1–2) with mean APACHE-II score of 14.2 (SD 4.68)) and 409 chronically ill patients (Supplementary Table 1).

In total, 2047 index and reference relatives were identified of which 665 (32.5%) were not registered in the general practice at the start of the follow-up period. Of 1382 relatives who were registered at the start of the follow-up period, a total of 35 (1.7%) lived in a household with more than one patient from the previous study and have, therefore, been excluded. No matched relatives were found for 375 (18.3%) participants, leaving a cohort of 267 index (mean age 38.1 (SD 23.6), 59.0% female) and 705 reference (mean age 36.3 (SD 23.6), 58.9% female) relatives. There were no significant differences in baseline characteristics between both groups (Table 1). A summary of the number of relatives in each timeframe is found in the supplemental data.

**Table 1.** Baseline characteristics of study population.

	Index relatives (*n* = 267)		Reference relatives (*n* = 705)		*p* Value
Age, years, mean (SD)^1^	38.1 (23.6)	*n* = 267	36.3 (23.6)	*n* = 705	.29^a^
Partners	54.5 (13.9)	*n* = 161	54.3 (12.8)	*n* = 406	.88^a^
Children	12.8 (7.7)	*n* = 106	11.7 (7.4)	*n* = 299	.21^a^
Partners, *n* (%)	161 (60.3)		406 (57.6)		.44^b^
Children, *n* (%)	106 (39.7)		299 (42.4)		
Male, *n* (%)	110 (41.0)		290 (41.1)		.98^b^
Female, *n* (%)	157 (59.0)		415 (58.9)		

ICU: intensive care unit; SD: standard deviation.

^1^ For relatives of ICU survivors, follow-up period started at ICU discharge of their relative. For relatives of matched reference, patients’ follow-up period started at ICU discharge of the ICU survivor to whom they are matched.

^a^ Calculated with independent samples *t*-test.

^b^ Calculated with chi-squared test.

### Total number of new episodes of care

No differences were found when comparing the number of new episodes in the timeframes within the first year after the start of the follow-up period. However, index relatives had a significantly higher number of new episodes 1–2 years (HR 1.26 per month, 95%CI 1.03–1.54) and 2–5 years (HR 1.28 per month, 95%CI 1.06–1.56) compared to the reference relatives (Table 2).

**Table 2.** Median [IQR] number of new episodes of care per month in each pre- and post-ICU timeframe compared to reference relatives.

Timeframe	Index relatives		Reference relatives		Hazard ratio (95%CI)^a^
–12 to 0 months	0.083 [0–0.25]	*n* = 267	0.083 [0–0.25]	*n* = 705	1.06 (0.90–1.25)
0–3 months	0 [0–0.33]	*n* = 267	0 [0–0.33]	*n* = 705	1.02 (0.87–1.19)
4–6 months	0 [0–0.33]	*n* = 267	0 [0–0.33]	*n* = 702	0.98 (0.80–1.19)
7–12 months	0 [0–0.24]	*n* = 264	0 [0–0.17]	*n* = 698	1.08 (0.88–1.33)
13–24 months	0.11 [0–0.25]	*n* = 245	0.08 [0–0.25]	*n* = 659	**1.26 (1.03–1.54)**
25–60 months	0.18 [0.06–0.31]	*n* = 211	0.13 [0.06–0.22]	*n* = 575	**1.28 (1.06–1.56)**

ICU: intensive care unit; CI: confidence interval.

Values presented as median [1st quartile–3rd quartile]. Hazard ratios in bold reached statistical significance at *p* < .05. Hazard ratios above 1 indicate significantly more new episodes of care in the index relatives. Hazard ratios below 1 indicate significantly more new episodes of care in the reference relatives.

^a^ Hazard ratios were calculated with a negative binomial regression analysis and were corrected for age, gender, general practice and early or late ICU discharge of former ICU patient.

### New episodes of care clustered by ICPC-2 chapters

Compared to the reference relatives, index relatives had a significantly higher number of new episodes for the digestive (0–12 months), hearing (13–24 months), circulatory (25–60 months), psychological (13–24 months), urinary (13–24 and 25–60 months), male genital (25–60 months) and pregnancy/childbearing (0–12 months) ICPC-2 chapters. Lower numbers were found in the index relatives for the endocrine/metabolic (0–12, 13–24 and 25–60 months) and pregnancy/childbearing (13–24 months, 25–60 months) ICPC-2 chapters. A difference in baseline was found between index and reference relatives in the urinary, social and metabolic/endocrine ICPC-2 chapters (Table 3).

**Table 3.** Hazard ratios (95%CI) for developing a new diagnosis in each pre- and post-ICU timeframe per ICPC-2 chapter compared to reference relatives.

	–12 to 0 months	0–12 months	13–24 months	25–60 months
A	1.16 (0.99–1.36)	1.13 (0.95–1.34)	0.98 (0.80–1.21)	1.18 (0.93–1.48)
B	0.99 (0.86–1.14)	0.97 (0.94–1.01)	1.03 (0.97–1.10)	1.03 (0.95–1.12)
D	0.97 (0.86–1.09)	**1.19 (1.03–1.37)**	1.01 (0.85–1.20)	1.02 (0.81–1.28)
F	1.07 (0.97–1.17)	0.90 (0.79–1.03)	1.10 (0.92–1.30)	1.19 (0.97–1.44)
H	1.03 (0.93–1.15)	0.92 (0.82–1.05)	**1.38 (1.18–1.62)**	**1.21 (1.00–1.47)**
K	0.98 (0.92–1.05)	1.02 (0.88–1.19)	1.05 (0.87–1.26)	**1.27 (1.02–1.56)**
L	0.92 (0.80–1.05)	1.03 (0.88–1.21)	1.12 (0.93–1.35)	1.23 (0.98–1.55)
N	1.20 (0.60–2.41)	0.98 (0.87–1.09)	1.05 (0.92–1.19)	1.00 (0.84–1.19)
P	0.94 (0.85–1.04)	0.90 (0.78–1.04)	**1.27 (1.07–1.51)**	1.07 (0.90–1.30)
R	0.94 (0.83–1.07)	1.02 (0.86–1.22)	1.10 (0.90–1.33)	1.25 (0.98–1.59)
S	1.01 (0.87–1.18)	0.98 (0.82–1.17)	1.04 (0.84–1.30)	1.18 (0.94–1.49)
T	**1.35 (1.26–1.44)**	**0.88 (0.81–0.95)** ^a^	**0.72 (0.66–0.79)** ^a^	**0.77 (0.68–0.86)** ^a^
U	**0.90 (0.83–0.99)**	1.04 (0.90–1.21)^a^	**1.35 (1.17–1.56)** ^a^	**1.22 (1.01–1.48)** ^a^
W	1.07 (0.96–1.20)	**1.33 (1.15–1.55)**	**0.71 (0.59–0.84)**	**0.71 (0.58–0.86)**
X	1.07 (0.93–1.23)	0.97 (0.84–1.11)	0.99 (0.84–1.16)	1.24 (0.96–1.58)
Y	1.13 (1.00–1.27)	0.95 (0.81–1.13)	1.03 (0.84–1.25)	**1.52 (1.10–2.10)**
Z	**1.17 (1.07–1.27)**	0.78 (0.67–0.91)^a^	1.02 (0.91–1.14)^a^	1.03 (0.88–1.21)^a^

A: general and unspecified; B: blood/ blood forming; D: digestive; F: eye; H: hearing; K: circulatory; L: musculoskeletal; N: neurological; P: psychological; R: respiratory; S: skin; T: endocrine/metabolic; U: urological; W: pregnancy/child bearing/family planning; X: female genital; Y: male genital; Z: social problems; ICU: intensive care unit; ICPC: International Classification of Primary Care.

Values are presented as hazard ratios (95% confidence interval). Hazard ratios were calculated with a negative binomial regression analysis and corrected for age and gender. Hazard ratios above 1 indicate significantly more new episodes of care in the index relatives. Hazard ratios below 1 indicate significantly more new episodes of care in the reference relatives. Hazard ratios in bold reached statistical significance at *p* < .05.

^a^ Corrected for age, gender and statistically significant difference in hazard ratio for developing a new diagnosis in the pre-ICU timeframe.

### New episodes of care clustered by ICPC-2 pathophysiology

Regarding the ICPC-2 pathophysiology chapters, index relatives had a significantly higher number of new episodes for the symptoms (13–24 and 25–60 months), infections (25–60 months), injuries (7–12, 13–24 and 25–60 months) and congenital (0–3, 4–6 and 13–24 months) chapters, and a lower number of new episodes for neoplasms (0–3, 7–12, 13–24 and 25–60 months) and the group of other diagnoses (4–6 months). A difference in baseline was found between index and reference relatives in the neoplasms, congenital and other diagnoses ICPC-2 pathophysiologies (Table 4).

**Table 4.** Hazard ratios (95%CI) for developing a new diagnosis in each pre- and post-ICU timeframe per ICPC-2 pathophysiology compared to reference relatives.

	–12 to 0 months	0–3 months	4–6 months	7–12 months	13–24 months	25–60 months
Symptoms	1.07 (0.91–1.27)	1.03 (0.91–1.17)	1.01 (0.84–1.22)	1.08 (0.88–1.31)	**1.33 (1.08–1.63)**	**1.31 (1.07–1.60)**
Infections	0.93 (0.80–1.07)	1.00 (0.90–1.10)	0.97 (0.86–1.10)	1.00 (0.84–1.18)	1.13 (0.94–1.35)	**1.25 (1.01–1.54)**
Neoplasms	**1.16 (1.09–1.23)**	**0.83 (0.79–0.89)** ^a^	1.01 (0.94–1.07)^a^	**0.84 (0.78–0.91)** ^a^	**0.84 (0.78–0.91)** ^a^	**0.83 (0.73–0.95)** ^a^
Injuries	0.97 (0.86–1.09)	0.97 (0.89–1.04)	0.93 (0.84–1.03)	**1.18 (1.06–1.33)**	**1.27 (1.10–1.47)**	**1.40 (1.15–1.70)**
Congenital	**0.89 (0.85–0.94)**	**1.11 (1.05–1.17)** ^a^	**1.07 (1.01–1.13)** ^a^	1.00 (0.95–1.06)^a^	**1.06 (1.00–1.12)** ^a^	1.00 (0.91–1.10)^a^
Other diagnoses	**1.19 (1.01–1.40)**	0.92 (0.75–1.11)^a^	**0.80 (0.65–0.97)** ^a^	0.86 (0.69–1.06)^a^	0.88 (0.70–1.10)^a^	1.06 (0.86–1.32)^a^

ICU: intensive care unit; ICPC: International Classification of Primary Care.

Symptoms: symptom diagnosis with a (yet) unknown origin. Other diagnoses include various diseases that do not fall under the other ICPC-2 pathophysiologies. Values are presented as hazard ratios (95% confidence interval). Hazard ratios were calculated with a negative binomial regression analysis and corrected for age and gender. Hazard ratios above 1 indicate significantly more new episodes of care in the index relatives. Hazard ratios below 1 indicate significantly more new episodes of care in the reference relatives. Hazard ratios in bold reached statistical significance at *p* < .05.

^a^ Corrected for age, gender and statistically significant difference in hazard ratio for developing a new diagnosis in the pre-ICU timeframe.

### Total number of consultations

Regarding the total number of consultations with the GP, no statistically significant differences were found between the index relatives and the reference relatives (Table 5).

**Table 5.** Hazard ratios (95%CI) for consulting the GP in each pre- and post-ICU timeframe compared to reference relatives.

	–12 to 0 months	0–3 months	4–6 months	7–12 months	13–24 months	25–60 months
All consultations	1.12 (0.87–1.43)	1.35 (0.94–1.95)	0.82 (0.54–1.25)	0.87 (0.58–1.29)	1.10 (0.72–1.68)	0.99 (0.67–1.48)

ICU: intensive care unit; GP: general practitioner.

Values are presented as hazard ratios (95% confidence interval). Hazard ratios were calculated with a negative binomial regression analysis and corrected for age, gender, general practice and early or late ICU discharge of former ICU patient. Hazard ratios above 1 indicate significantly more consultations in the index relatives. Hazard ratios below 1 indicate significantly more consultations in the reference relatives.

## Discussion

### Main findings

This study showed that relatives of ICU survivors had a higher number of new episodes in primary care from the second to the fifth year after ICU discharge of their relative than the relatives of chronically ill patients in the same period. There was an excess in new episodes of 26% in the second year and 28% in the third to fifth year.

### Strengths and limitations

This is the first study that uses ‘real life’ data of a primary care population to study new episodes in relatives of ICU survivors. In the Dutch healthcare system, GP data comprise all new episodes that primary care and secondary care giving a complete overview of all health problems that led to professional interference. Furthermore, our data were extracted from a registration network of which the quality and accuracy of registration of diseases and diagnoses is subject to continual quality control [20–22].

No restrictions in the inclusion of new episodes were set while other studies mostly focus on certain PICS-F related symptoms, such as mental and cognitive health problems [10,13–15]. This gave us a broad view of health problems of relatives of ICU survivors in primary care.

Although we performed our analysis in a historical cohort, the data, including the ICPC-2 codes, from our research network are registered prospectively at the time of patient encounter, making detection bias unlikely.

A limitation is that the secondary outcomes did not allow firm conclusions about the difference in health status between the index and reference group due to multiple testing and relatively low numbers per ICPC-2 chapter. These outcomes should be seen as exploratory and hypotheses-generating for further follow-up studies. Furthermore, although both groups had similar health demands prior to the event, some ICPC-2 chapters showed a slight difference in baseline. As a result, these baseline differences might have led to respectively more or less new episodes of care in the post ICU timeframes. For example, Table 3 shows more new endocrine/metabolic (T) episodes in the pre-ICU time frame in the ICU group, which may result in less new T-episodes afterwards.

Moreover, we could not correct for the differences in the burden or impact of the disease from which the included critically ill (index group) and chronically ill (reference group) patients suffer. A longer ICU stay or a more severe chronic illness may have a higher burden on relatives but it could not be studied with our data.

### Comparison with existing literature

Contrary to our findings, several studies reported that health problems of relatives of ICU patients were most prominent in the first year after ICU discharge [10,14,15,26]. In our study in primary care, however, no statistically significant difference was found in the overall number of new episodes and in the matching ICPC-2 chapters (psychological, musculoskeletal, neurological and cardiovascular) in the first year after ICU discharge. This does not prove that such complaints do not occur in relatives of ICU patients the first year after discharge; instead it may show that relatives do not seek professional help in case complaints develop. Moreover, this difference might be explained by the fact that other studies actively approached relatives of ICU survivors with questionnaires and interviews and did not rely on help-seeking behaviour in primary care. Literature states that patients suffer from significantly more symptoms than they present to a physician [27]. Furthermore, relatives may prioritise the health of the ICU survivor over theirs and therefore are reluctant to consult the GP the first months after ICU discharge. Or maybe they received mental support through the healthcare provider of their relative. This creates a time-lag, which may also explain why we found an excess in new episodes in 2–5 years after ICU discharge.

The finding that relatives of ICU survivors developed more new episodes than relatives of chronically ill patients in the long term (2–5 years) is striking. It is known that depressive symptoms occur and may last longer than one year after discharge of the relative [13]. Hence, our findings of excess in morbidity (i.e. new episodes) more than one year after discharge might result from chronic psychological distress leading to help-seeking behaviour for a broad range of symptoms and diseases; this is hypothetical, however. It should be noted that it is known that relatives of chronically ill patients (not necessarily ICU survivors) report a wide array of health problems an average of seven years after the diagnosis of their relative [28]. As we compared with a reference group of relatives of chronically ill patients, our data demonstrate that having a relative on the ICU might be an extra burden on top of having a chronically ill relative.

### Implications for practice

Since our study was exploratory and hypothesis-generating, our findings and theories regarding our results are hypothetical and warrant further research. This study might highlight the need for awareness of health problems relatives of ICU survivors. Our findings might suggest that these relatives prioritise the health of the ICU survivor over their own in the first year after ICU discharge, which may lead to a deterioration in health and excess in new health problems after this first year. We hope our research might lead to an improved understanding of the effects of ICU admission on relatives of ICU survivors among GPs and GP staff. A short notification in the patients’ medical files whose relative survived the ICU might enhance awareness. Moreover, it might be beneficial that this group receives extra support or care from their GP. For example from family-based models of care (e.g. combined GP consultations with ICU survivor and relative, illness-specific education), along with cognitive behavioural therapy and increased social support for the relatives who need such help [29]. Especially, in this time during the COVID-19 epidemic in which we see a stark increase in ICU admissions, which might lead to an increase in PICS-F related health problems.

## Conclusion

This study showed that relatives of ICU survivors have significantly more new episodes in primary care in the second to fifth year after ICU discharge of their relative compared to relatives of chronically ill patients.

## Supplementary Material

Supplemental Tables and Figures
