# Supplementary material for: The impact of an intensive care unit admission on the health status of relatives of intensive care survivors: A prospective cohort study in primary care
Source: Eur J Gen Pract. 2022 Apr 7;28(1):48–55. doi: 10.1080/13814788.2022.2057947 (PMC9004533; doi:10.1080/13814788.2022.2057947)
Supplement: Supplemental Tables and Figures [file IGEN_A_2057947_SM7129.docx]

**Supplemental Tables and Figures**

| Table S1. Baseline characteristics of former ICU patients and chronically ill patients. | | | | | |
| --- | --- | --- | --- | --- | --- |
|  | Former ICU patients (n = 153)  (27.2%) | | Matched chronically ill patients  (n = 409) (72.8%) | | p-value |
| Age at start follow-up period, years, mean (SD) | 56.9 (14.2) | | 55.5 (13.6) | | .270 ^a^ |
| Gender (male), n (%) Gender (female), n (%) | 107 (69.9) 46 (30.1) | | 287 (70.2) 122 (29.8) | | .956  ^b^ .956  ^b^ |
| Pre-existing comorbidity, n (%) ^1^  None  Neoplasm  Cardiovascular  Infection  Psychological  Skin  Respiratory  Congenital  Other | 5 (3.3%)  50 (32.3%)  109 (71.2%)  1 (0.7%)  50 (32.3%)  16 (10.5%)  34 (22.2%)  3 (2.0%)  124 (81.0%) | n = 153 | 26 (6.4%)  112 (27.4%)  272 (66.5%)  0 (0%)  109 (26.7%)  46 (11.2%)  82 (20.0%)  3 (0.7%)  323 (79.0%) | n = 409 | .153 ^b^  .217 ^b^  .285 ^b^  .102 ^b^  .158 ^b^  .790 ^b^  .571 ^b^  .208 ^b^  .588 ^b^ |
| Reason for ICU admission: surgical/ other, n (%)  Surgical  Other | 118 (77.1%) ^4^ 35 (22.9%) | n = 153 | NA |  |  |
| ICU stay, days, median [1^st^ quartile-3^rd^ quartile] | 1 [1-2] | n = 125 ^2^ | NA |  |  |
| Mechanical ventilation, days, median [1^st^ quartile-3^rd^ quartile] | 1 [1-2] | n = 92 ^2^ | NA |  |  |
| APACHE-II score, mean, (SD) ^3^ | 14.15 (4.68) | n = 98 ^2^ | NA |  |  |
| ICU re-admissions, n (%)  0  1  >1 | 131 (85.6%)  15 (9.8%)  7 (4.6%) | n =153 | NA |  |  |
| *ICU: Intensive care unit; SD: Standard deviation; IQR: Interquartile range; NA: not applicable. APACHE-II: Acute physiology, age and chronic health evaluation II.*  *^a^ Calculated with independent samples t-test.*  *^b^ Calculated with chi-squared test.*  *^1^ Pre-existing comorbidity was considered present if it was registered before hospital discharge following first ICU admission. For chronically ill reference patient’s comorbidities registered prior to the ICU discharge of the matched ICU patient were used.*  *^2^ Reduced number due to missing data.*  *^3^ A numerical severity of disease classification system, applied within 24 hours of admission of a patient in the ICU. Used to generate a score between 0 and 71; higher scores correlate with higher ICU mortality* (28)*.*  *^4^ Skewed distribution due to Radboudumc being a center for cardiothoracic surgery which leads to more ICU stays after elective cardiothoracic surgery.* | | | | | |


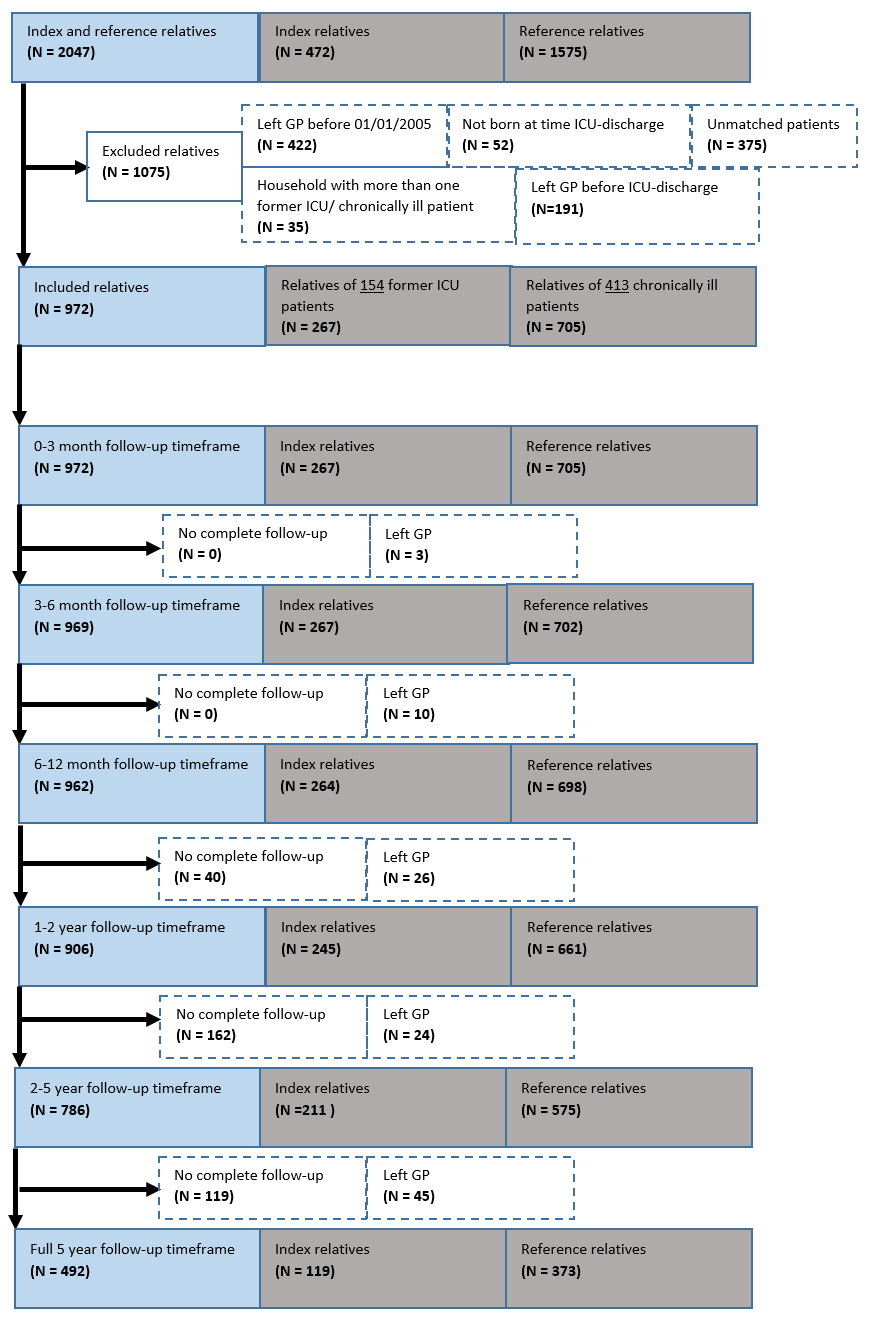


**Figure 2.** Flowchart of study population including index relatives and reference relatives. No complete follow-up refers to patients of which the duration of the follow-up period to the end of the inclusion period (01/07/2019) was shorter than the duration of a certain timeframe.
